# Concurrent Validity and Reliability of Three Ultra-Portable Vertical Jump Assessment Technologies

**DOI:** 10.3390/s20247240

**Published:** 2020-12-17

**Authors:** Casey M. Watkins, Ed Maunder, Roland van den Tillaar, Dustin J. Oranchuk

**Affiliations:** 1 Sports Performance Research Institute New Zealand, Auckland University of Technology, 1142 Auckland, New Zealand; casey.watkins@aut.ac.nz (C.M.W.); ed.maunder@aut.ac.nz (E.M.); dustin.oranchuk@aut.ac.nz (D.J.O.); 2 Department of Sports Sciences and Physical Education, Nord University, 7601 Levanger, Norway

**Keywords:** force-plate, motion-capture, reactive strength index, stiffness, variability

## Abstract

Vertical jump is a valuable training, testing, and readiness monitoring tool used across a multitude of sport settings. However, accurate field analysis has not always been readily available or affordable. For this study, two-dimensional motion capture (Mo-Cap), G-Flight micro-sensor, and PUSH accelerometer technologies were compared to a research-grade force-plate. Twelve healthy university students (7 males, 5 females) volunteered for this study. Each participant performed squat jumps, countermovement jumps, and drop jumps on three separate occasions. Between-device differences were determined using a one-way repeated measures ANOVA. Systematic bias was determined by limits of agreement using Bland–Altman analysis. Variability was examined via the coefficient of variation, interclass correlation coefficient, and typical error of measure. Dependent variables included jump height, contact-time, and reactive strength index (RSI). Mo-Cap held the greatest statistical similarity to force-plates, only overestimating contact-time (+12 ms). G-Flight (+1.3–4 cm) and PUSH (+4.1–4.5 cm) consistently overestimate jump height, while PUSH underestimates contact-time (−24 ms). Correspondingly, RSI was the most valid metric across all technologies. All technologies held small to moderate variably; however, variability was greatest with the G-Flight. While all technologies are practically implementable, practitioners may want to consider budget, athlete characteristics, exercise demands, set-up, and processing time before purchasing the most appropriate equipment.

## 1. Introduction

The value of vertical jumping is well-established for training and testing across many sports and exercise settings [1,2]. Demonstrating large correlations with sprint speed (r = 0.70–0.91), the ability to absorb and redirect force is a primary consideration in many team sports and track and field events [3]. Thus, understanding exercise parameters, such as jump height and contact times, allows coaches to manipulate explosive multi-joint exercises to train many different adaptations, including speed, power, reactive strength, force absorption, and proprioception [4,5]. However, a key factor for coaches using jump exercises for training and testing is the accuracy of these measurements.

There are several methods available to monitor jump performance spanning from a ruler and a wall to the most expensive advanced clinical technologies. Traditionally, a stand-and-reach test (Vertec) measuring center of mass displacement from subjects swatting aside plastic vanes has demonstrated large biases, both over-estimating jump height by 11.2 cm versus the Optojump [6], and under-estimating jump height by 2.4 ± 6.6 cm (mean ± standard deviation) compared to force-plates [7]. This typically occurs due to the set-up variability within an athlete’s arm swing, wherein more or less arm involvement can drastically affect the jump height reached. As a result, newer methodologies encourage static hand placement, either gripping a wooden dowel or placed akimbo. Research-grade multiaxial force-plates are generally considered the “gold standard” for accurately measuring ground reaction forces created during jumping due to multiple inlaid sensors, high capacities, and increased sensitivity. However, newer technologies, including linear position transducers, mobile phone-based applications, micro-lasers, and accelerometers, are attempting to more easily and affordably bring accuracy to the practitioner [8].

Typically, several factors can influence the device’s accuracy, including the measurement method, sampling rate, testing area, athlete characteristics, instrument set-up, verbal instructions, and post-production algorithms. Jump height calculations based on displacement and take-off velocity have traditionally been the most accurate, but many newer technologies have reported high validity using flight time methods instead [9]. Accelerometers use small inertial sensors to measure velocity, while linear position transducers use manual cable movement to measure vertical displacement [10]. Both can be mounted directly on the athlete’s body via a belt or band or on a held bar. Therefore, instrument set-up, sensor placement, and anterior-posterior trunk deviations may consequently affect measurement accuracy [11]. For accelerometers, the inclusion of a gyroscope may account for landing position and trunk inclination [11], while linear transducers are advised to be set-up as vertical as possible. On the other hand, camera-based apps and micro-lasers use flight time primarily to calculate jump height, and therefore rely on proper jump and landing mechanics for accuracy [12]. The main difficulty with body-mounted accelerometers and camera-based applications is the correct identification of take-off and landing [13]. While camera-based applications rely on manual frame selection to determine take-off and landing [10], body-mounted accelerometers use automatic algorithms. However, some authors have advised similar lengthy manual identification methods are necessary to reduce systematic bias in body-mounted accelerometers [13].

While the validity and reliability of several devices have been examined, several common limitations exist. For example, most studies only examine homogenous subjects and jumps, making it difficult to ascertain accuracy with both good and poor performers [14,15]. Most studies have utilized only a few statistical strategies, including limits of agreement (LoA), coefficient of variation (CV), intraclass correlation coefficient (ICC), Pearson’s r, and/or *p*-values [15,16], which raises some issues. For instance, the most commonly reported reliability statistics, the ICC and r, are dependent on between-subject variability, which minimally affects typical error of measure (TEM) and CVs [17,18]. Similarly, *p*-values only assess systematic bias between measures and are largely dependent on sample size [18]. Additionally, whilst systematic bias gives important information on whether a specific data-point is likely to be an under- or over-estimation compared to the criterion gold standard, it does not provide insight into the reliability of an estimate. Reliability is also often derived from a single session (i.e., within-trial variation), which has limited application to test-retest methodologies [8,14,15,19]. Finally, while several studies have examined the validity and reliability of force-plates, motion capture, and accelerometry-based technologies [13,14,20], the G-Flight photo-cell system, an affordable and extremely portable means of assessing jump performance, has yet to be independently validated. Therefore, the primary aim of this investigation was to compare the validity and reliability of estimated jumping performance from three portable commercially available products (G-Flight, PUSH, two-dimensional motion capture) simultaneously against a criterion laboratory-grade force-plate. Specifically, the authors aimed to address differences in absolute measurement output, intra- and inter-session variation, and composition of measurement error between portable devices compared to the force-plate. In doing so, we aimed to provide practical information for coaches to determine which technology is best suited for their needs. We hypothesized that the G-Flight would hold strong validity and reliability, like other laser-based technologies (e.g., Optojump), while the PUSH would be the least accurate and reliable of the four products due to the added difficulty for manufacturers to develop sufficient algorithms to estimate take-off and landing.

## 2. Materials and Methods

### 2.1. Experimental Design

Using a repeated measures design, jump height, contact-time, and the reactive strength index (RSI) were collected via force-plate (AMTI), two-dimensional motion capture (Mo-Cap), a micro-laser timing system (G-Flight), and body-mounted accelerometry (PUSH). Each participant was tested on three occasions, separated by five to eight days. The differences in measurement outputs of the technologies were determined via analysis of variance (ANOVA), while CV, ICC, and TEM were utilized to determine variability. Finally, LoAs were calculated to determine systematic bias.

### 2.2. Subjects

Twelve healthy university students (7 males, 5 females) (age = 28 ± 2.4 years, height = 165.2 ± 17.7 cm, mass = 82.3 ± 34 kg) volunteered. All subjects were free of musculoskeletal injuries in the three months before data collection and required to be participating in some form of physical activity at least twice weekly. Subjects were instructed to maintain their current level of physical activity throughout the data collection period, though this was not specifically tracked. The Auckland University of Technology Research Ethics Committee approved the study (17/422), and all subjects gave informed consent before study involvement.

### 2.3. Testing Procedures

**Figure 1:**
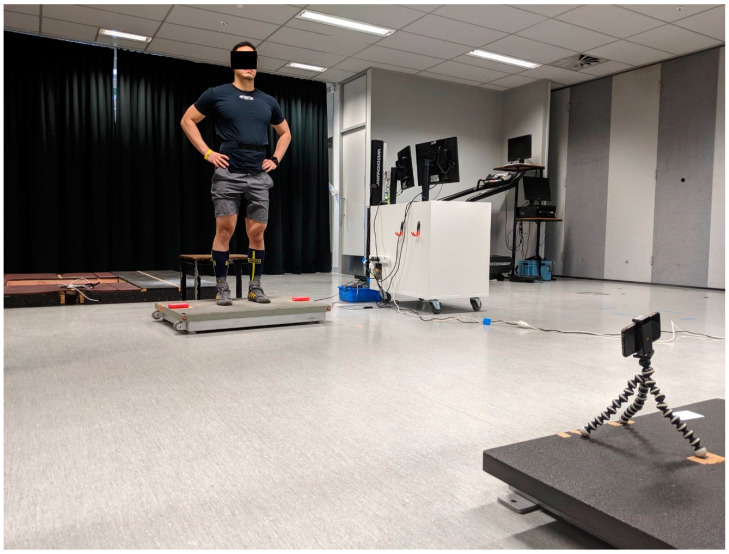
Testing equipment set-up.

Upon arriving for each testing session, the subjects performed a standardized warm-up, as detailed previously [21]. After which each participant performed, three squat-(SJ), countermovement-(CMJ), and drop-jumps (DJ), in that order. The order of jump types was not randomized to minimize any differences between sessions and improve the overall reliability of testing. Rest between repetitions and between jump types was set at 30 and 60 s, respectively. All jumps were performed with the hands-on-hips to improve control and reduce the variability of arm swing. Encouragement to jump as high as possible was provided by the same researcher each jump. During the SJ, subjects descended to a knee angle of approximately 90° [22], and held this position for at least two seconds before explosively propelling themselves vertically. Trials were checked carefully to prohibit a countermovement action. The CMJs were performed with a rapid descent to a self-selected depth, immediately followed by a maximal ascent [1,22]. DJs were performed from a height of 20 cm (30 cm above the 10-cm force plate; Figure 1). The subjects were instructed to drop from the box, “attack the ground”, and land with stiff ankles and knees to minimize ground contact-time and simultaneously maximize jump height. A jump was considered successful if the athlete gave maximal effort, the hands did not leave the hips, and there was no obvious front-to-back or side-to-side displacement. Particularly for the G-flight, foot placement was visually checked to ensure a similar contact area of the foot between the laser line on take-off and landing. Additionally, all jumps were visually checked for any modifications that would allow for greater performance. These modifications included a pre-jump backwards sway or vertical motion, forced hip flexion, or excessive knee bending prior to ground contact. If the criteria for a successful jump were not met, a maximum of three additional jumps were allowed. As the primary purpose of the study was to examine concurrent reliability, jump types were not randomized. The inclusion of both sexes and three jump types were to create a larger range of jump heights, contact times, and RSIs.

Four technologies simultaneously collected all jumps (Figure 1). Jumps were performed on a force-plate (AMTI, Watertown, MA, USA) sampling at 1000 Hz, interfaced with a custom LabView software (National Instruments, Austin, TX, USA). Mo-Cap data were collected via a mobile phone (iPhone 8, Apple Inc., Cupertino, CA, USA) recording at 240 frames-per-second in full HD (1920 × 1080 pixels) The phone was stationed on a tripod positioned 3 m to the front, and 15 cm above the force-plate. The G-Flight timing system (Exsurgo, Sterling, VA, USA), sampling at 32,000 Hz, was placed at each side of the force-plate, 62 cm apart. The sensors were positioned as recommended by the manufacturer. Subjects were instructed to stand with both feet positioned between the G-Flight units with the hallux of the outermost toe in line with the laser. Finally, the PUSH 2.0 accelerometer (PUSH Inc., Toronto, ON, Canada) was strapped to the lower back of each participant, and sampled at 200 Hz.

### 2.4. Data Processing and Analysis

Flight time was used to calculate jump height for all four technologies. Force-plate data were saved following each jump and processed offline using a custom MATLAB code to calculate flight and ground contact-time (MathWorks, Natick, MA, USA). For all jump types, body weight (BW) and the SD of BW were calculated from the average of the first 500 ms of unfiltered data in the *Z*-axis. For peak concentric and landing forces, the tri-axial (X, Y, Z) data were processed through a 10 Hz Low Pass 4th order Butterworth filter. Both peak forces were then identified (Z > 130% BW). Subsequently, for SJ, the onset of movement was identified from the point before concentric peak force where Z > BW. Whereas, for CMJ, the onset of movement is defined as the point at which force drops five SD below BW. If no movement was detected from 5 SD below, then 3 SD was used. For DJ, the athlete stands on the force plate to obtain BW like other jumps; then, while the force plate is still collecting steps onto the box, standing on the box for ~0.5 s to obtain a clear absence of force. The onset of movement is defined as the point at which Z > 20 N after 1.5 s. The take-off point was identified for all three jump types as the first point after peak concentric force and before peak landing force where Z < 20 N; landing was considered the last point of this zero-force period prior to the peak landing force. Jump height was then calculated off flight-time via the formula 9.81 × (flight time)^2^/8 [23]. This calculation was consistently used for all measurement devices.

Mo-Cap video footage was loaded into Kinovea 0.8.15 motional analysis software and manual frame-by-frame identification of each jumping phase was performed by a researcher. Flight time was defined as the time at the final frame when the participant is clearly in contact with the force-plate during the propulsive phase of the jump, minus the time of the frame where the participant re-contacts the force-plate. Similarly, the contact-time was defined as the time of the first frame where the participant contacts the force-plate after dropping from the box, until the time of the last frame before leaving the force-plate. Similar two-dimensional motion capture and Kinovea 0.8.15 software is highly valid and reliable (r = 0.99) for assessing high-velocity movements [20]. Flight time was converted to jump height via the formula 9.81 × (flight time)^2^/8 (5). RSI was calculated by dividing jump height in centimeters, by contact-time in milliseconds [16]. The G-Flight photocells estimate contact-time as the period where the laser is broken by the participant and flight time as the period where the laser is unbroken. G-Flight readings of jump height, contact-time, and RSI were manually recorded following each jump and subsequently transferred to an Excel (version 2016; Microsoft Corporation, Redmond, WA, USA) spreadsheet. The PUSH accelerometer was connected via Bluetooth to a smartphone (iPhone 8, Apple Inc., Cupertino, CA, USA) application (Train with PUSH, Software version 4.5.0). Following each session, the “PUSH portal” was accessed and PUSH data were exported to an Excel spreadsheet.

### 2.5. Statistical Analysis

To compare the validity of jumping heights, contact-times, and RSI between the four devices and testing time, a 3 (test session 1–3) × 4 (device) repeated measures analyses of variance (SPSS Statistics, v25, SPSS Inc., Chicago, IL, USA) was used. The Shapiro–Wilks test was used to determine the distribution of the data. Where the sphericity assumption was violated, the Greenhouse–Geisser-corrected *p*-values in the results were reported. Post-hoc comparisons were performed by using Holm–Bonferroni stepwise correction between Mo-Cap, G-Flight, and PUSH devices and the corresponding criterion force-plate measurement. The statistical significance level was set at *p* < 0.05. Additionally, the magnitude of the difference was assessed by effect sizes using η_p_^2^ (partial ETA squared), where 0.01 < η_p_^2^ < 0.06 constitutes a small effect, 0.06 < η_p_^2^ < 0.14 constitutes a medium effect, and η_p_^2^ > 0.14 constitutes a large effect [24]. Percent difference and Cohen’s d effect sizes (ES) with 95% confidence intervals (95% CI) were calculated relative to force-plate outputs. ESs were assessed using these criteria: trivial < 0.2, small = 0.2–0.49, moderate = 0.5–0.79, large > 0.8 [25]. The level of agreement between permutations of jump performance measured via Mo-Cap, G-Flight, and PUSH devices and the corresponding criterion force-plate measurement was made using Bland-Altman 95% LoA [26]. Typical error of measurement (TEM), which was measured by the average standard deviation per test session for each subject, was used to assess the typical error in the measurements and Bland–Altman plots to identify potential systematic bias, which was reported through mean-bias and standard deviations [27].

To assess the within-session reliability of the three repetitions of each set with each measuring device, the ICC (3,1) and the coefficient of variation (CV) for each test session were used. To compare the reliability (CV and TEM) over the three sessions of jumping heights, contact-times, and RSI between the four devices and testing time, a 3 (test session 1–3) × 4 (device) repeated measures ANOVA was used. The thresholds for interpreting ICC results were: 0.20–0.49 low, 0.50–0.74 moderate, 0.75–0.89 high, 0.90–0.98 very high, and ≥0.99 extremely high. The average within-session reliability of each measure was interpreted as acceptable for an ICC ≥ 0.67 and a CV ≤ 10%, moderate when ICC < 0.67 or CV > 10%, and unacceptable/poor when ICC < 0.67 and CV > 10% [28,29,30]. The force-plate was considered the criterion technology.

## 3. Results

Means, standard deviations, minimum and maximum values, percentage, and ES (with 95% CI) difference relative to force-plate for each dependent variable, and measurement technology are presented in Table 1.

### 3.1. Validity

**Figure 2:**
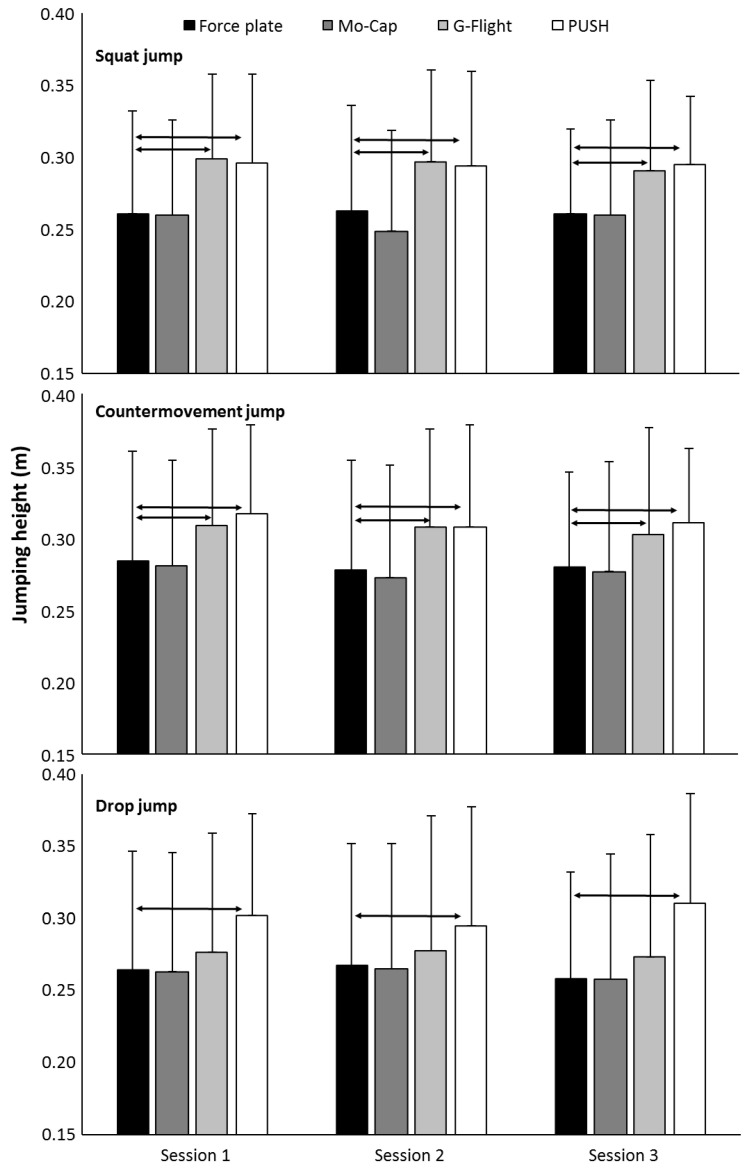
Mean (±SD) jump height per session recorded by force-plate, Mo-Cap, G-Flight, and PUSH across squat, countermovement, and drop jumps. Arrows denote *p* < 0.05.

**Figure 3:**
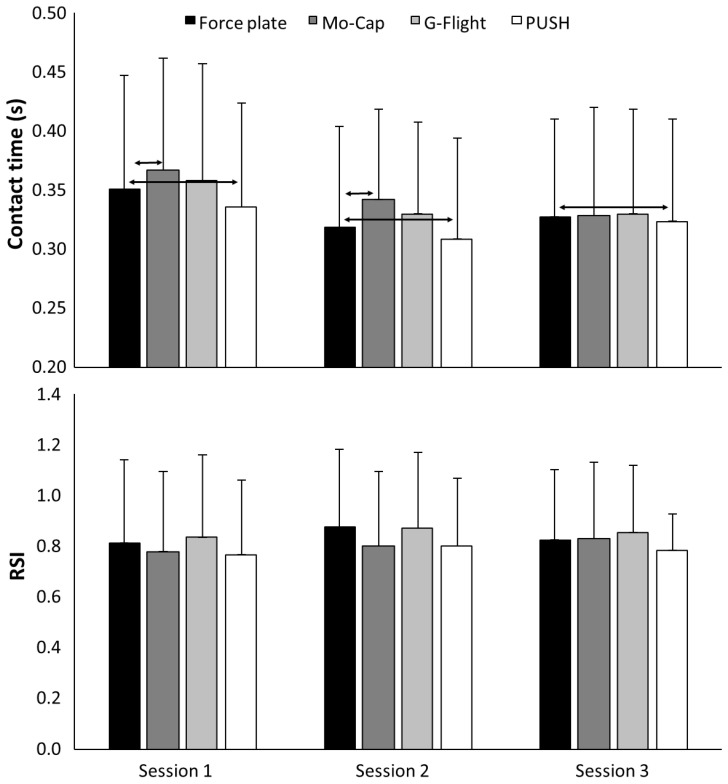
Mean (±SD) contact-time and reactive strength index per session recorded by force-plate, Mo-Cap, G-Flight, and PUSH across squat, countermovement, and drop jumps. Arrows denote *p* < 0.05.

**Figure 4:**
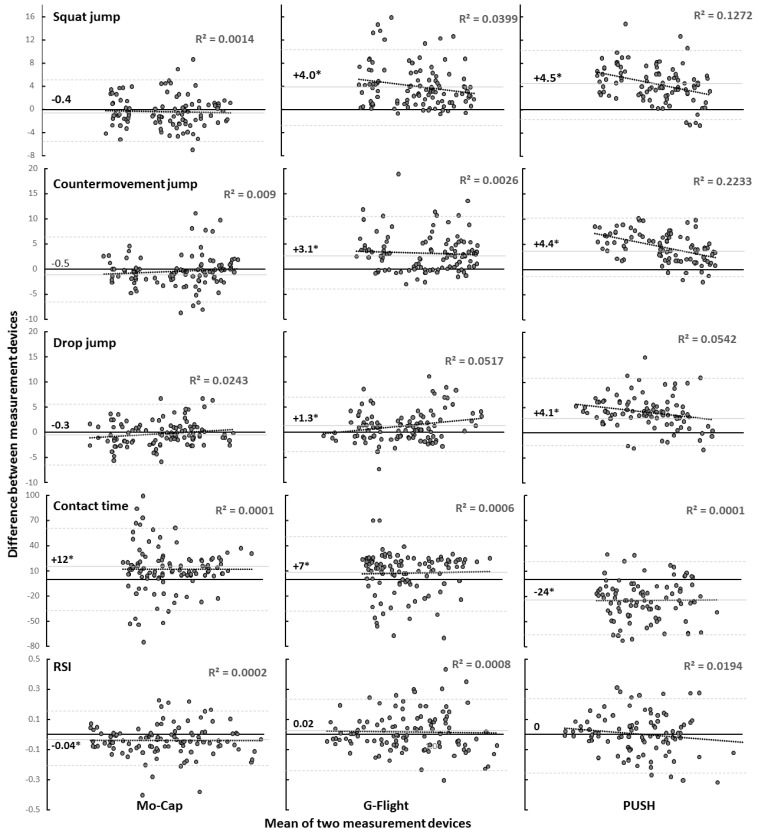
The difference in the mean jump performance measures between force-plate and Mo-cap, G-Flight, and PUSH.

All data were normally distributed (*p* > 0.05). No significant difference in SJ, CMJ or DJ height, contact-time, or RSI was found over the three test sessions for any of the devices (F ≤ 0.89, *p* ≥ 0.43, η_p_^2^ ≤ 0.11). Furthermore, no interaction effect (F ≤ 1.8, *p* ≥ 0.186, η_p_^2^ ≥ 0.07) was present. However, a significant effect of measuring device was found for jump height (F ≥ 21, *p* < 0.001, η_p_^2^ ≥ 0.68) and contact-time (F = 26.9, *p* < 0.001, η_p_^2^ = 0.73) but not for RSI (F = 1.6, *p* = 0.21, η_p_^2^ = 0.14). Pairwise comparisons for jump height are illustrated in Figure 2, with pairwise comparisons of contact time and RSI illustrated in Figure 3. Post-hoc comparisons revealed both the PUSH band and G-Flight measured greater jump heights than the force-plate for the SJ and CMJ; however, only the PUSH band measured greater values across all sessions for the DJ (Figure 2). Both the G-Flight (+2.8 ± 3.6 cm) and the PUSH (+4.3 ± 3.1 cm) calculated greater jump heights than the force-plate (*p* < 0.001). Contact-times were significantly longer measured with the Mo-Cap (+12 ± 28 ms, *p* < 0.01) and G-Flight (*p* = 0.002) tools, while contact-times were significantly shorter when measured with the PUSH (+7 ± 24 ms, *p* < 0.01) compared with the force-plate. LoA-derived systemic bias was flat, with the three largest values for PUSH-estimated CMJ height (4.4 cm, r^2^ = 0.223), SJ height (4.5 cm, r^2^ = 0.127), and DJ height (4.1 cm, r^2^ = 0.054), respectively (Figure 4).

### 3.2. Reliability

No significant differences between the three test sessions were found for TEM (F ≤ 1.7, *p* ≥ 0.20, η_p_^2^ ≤ 0.18) or CV (F ≤ 1.6, *p* ≥ 0.24, η_p_^2^ ≤ 0.29) for any of the jump heights, contact-time, or RSI. However, significant main effects of the measurement device were found for CMJ and DJ height, and RSI (F ≥ 3.2, *p* ≤ 0.034, η_p_^2^ ≥ 0.25) but not SJ height or contact-times (F ≤ 0.3, *p* ≥ 0.81, η_p_^2^ ≤ 0.05). In addition, no significant device*test session interaction effect was found for TEM and CV for any of the variables (F ≤ 1.7, *p* ≥ 0.123, η_p_^2^ ≤ 0.20). The post-hoc comparison revealed that the TEM and CV were significantly higher for CMJ and DJ height, and RSI for the G-Flight than the force-plate in most test sessions, while the TEM of the CMJ of the Mo-Cap and PUSH only were significantly higher in one session (Table 2). The CV for the CMJ was also only higher in one session for the Mo-Cap device compared with the force-plate (Table 2). The CV for all jump heights and contact-times for all devices was below 9%. However, the CV for the RSI occasionally surpassed 10% with the Mo-Cap, G-flight, and PUSH system. The ICCs of all measurements were all very high or extremely high (Table 2).

## 4. Discussion

The main purpose of this investigation was to examine the validity and reliability of commercially available portable technologies against lab-grade research force-plates. For jump height, both G-Flight and PUSH overestimated the maximal height by ~4 cm, while no difference in maximal height was observed between Mo-Cap and force-plate calculations. Contact-times were significantly longer and shorter than the force-plate for Mo-Cap and PUSH, respectively. RSI was not significantly different between devices. In general, variability was higher for CMJ and DJ heights when measuring with the G-Flight compared with the force-plate but still fell into the ‘acceptable’ range (ICC > 0.67, CV < 10%).

These results are similar to other publications reporting trivial overestimations (0.25–1.8 cm) using more automated 2-D motion capture apps like MyJump [8,10], compared to force-plates. In comparison, G-Flight and PUSH calculations revealed significant overestimations for SJ and CMJ heights (+3–4.5 cm). Alternatively, only PUSH overestimated jump height when performing the DJ (+4.1 cm). Wee et al. (2018) similarly reported overestimations (though somewhat larger) of 14.4 cm with PUSH, and 12 cm with GymAware to force-plates. Differences in the magnitude of overestimation are most likely due to sensor placement, with the upper spinal placement likely to increase the error due to trunk inclination compared to the lumbosacral position used in the current. It is also important to note that the LoA for all devices, jump measures, and jump heights were very flat (r^2^ = 0.0002–0.223) (Figure 4), indicating that little to no systematic biases were present between high (≤48.5 cm, ≥191 ms, RSI ≤ 1.50) and low (≥8.7 cm, ≤584 ms, RSI ≥ 0.26) performing jumpers (Table 1). Therefore, researchers and practitioners can utilize any of the examined technologies across a wide range of subjects, if it is understood that the devices are generally not interchangeable. However, it should also be noted that PUSH-derived jump heights were the three largest biases, suggesting that PUSH may not be the best choice when assessing changes in jump height due to training or acute fatigue.

To the authors’ knowledge, there have been very few published studies that have analyzed variance in jump heights, contact-time, or RSI in different measurement devices over three jump types. Furthermore, this is the first study examining the G-Flight micro-sensor system. Mo-Cap, G-Flight, and PUSH generally held similar testing variability as the force-plate. However, while ‘acceptable’, the G-Flight was commonly significantly more variable than the force-plate (Table 2). However, this increased variability does not preclude the use of the G-Flight so long as practitioners understand that a larger shift in jump performance will be required before they can be sure a real change has occurred. Therefore, it is recommended that practitioners calculate the smallest worthwhile change from the results, or for their specific tests and populations.

There are some limitations to the present study. Firstly, to standardize jump techniques, and minimize repetitions with large forward to backward displacement (where the G-Flight laser would not be tripped), jumps performed with arm-swing were not included, limiting the maximal jump heights examined. Therefore, future studies should examine the validity and reliability of very high jumps. While purely anecdotal, it is plausible that the G-Flight consistently overestimated jump height due to an occasional forward displacement during jumps, combined with the toes contacting the force-plate before the heel. Similarly, the G-Flight could be tripped by the removal of the midfoot a few milliseconds before toe-off. As such, the G-Flight micro-sensor could be tripped slightly before or after the other technologies. Likewise, it is important to note that all jump metrics were calculated using flight time, and not take-off velocity via the impulse momentum method, a decision made to ensure a fair comparison between devices. However, readers should be aware of the inherent issues with flight time calculations, including landing with excessively flexed knees and hips. Randomizing jump types and including extremely high and low performers could have been beneficial to the study. Furthermore, examining jumps with arm-swings would improve ecological validity. Thus, it is recommended that future studies utilizing the G-Flight instruct subjects to land flat-footed. It should also be recognized that while precedent exists for the specific variability cut-offs in the present study [29,30], no universal consensus exists [17,18,19]. Therefore, practitioners may wish to apply their own inference scales.

Athletes, practitioners, and researchers can apply the findings of the present investigation in several ways. Depending on variables of interest, time, money, and practical application, all technologies can be practically implementable. While Mo-Cap was the most valid and affordable technology, it also involves the greatest processing time. For teams with a small support team or many athletes, the added hours of analysis time may not be practical on a consistent basis. G-Flight slightly overestimated CMJ and SJ height and held the greatest variability but was both valid and reliable for contact-time and RSI. Practically speaking, this technology was more accurate for time-sensitive metrics but was significantly more variable than the force-plate for jump height between sessions. This is unsurprising considering G-flight is solely based on flight time, and varying movement strategies will produce a large variance in jump height for similar contact times. Since G-flight only offers a small number of variables, analysis of movement strategy is near impossible. However, quick processing time and ease of set-up make this a good tool for testing large groups efficiently, although, some familiarization is may be necessary to ensure proper foot placement and landing cues. Moreover, with novice athletes or athletes with little jumping experience, jump height variation may be exemplified, and thus coaches will need a larger shift in performance to account for wider CV. For weekly readiness or monitoring needs, this software may not be sensitive enough for minute changes, whereas changes across weeks or months may be more easily recognized. PUSH held moderate over- and under-estimations for jump height and contact-time, respectively, but allow analysis for a myriad of different exercises. However, the cost of each additional unit or having to swap athlete set-ups mid-exercise makes testing large groups difficult, making this technology more beneficial for in-depth analysis across individuals and small groups. Practitioners should think about the number of athletes, available processing time, athlete experience, and exercise demands before purchasing available equipment.

## 5. Conclusions

The present study demonstrates the utility of all four vertical jump assessment technologies. Mo-Cap and force-plates were the most similar. In comparison, G-Flight and PUSH tended to overestimate jump heights, while PUSH underestimated contact-times. However, all technologies reported nearly identical RSI estimates. Researchers and practitioners should be cognizant of validity and reliability, and the convenience and portability of each of the assessed technologies before purchasing or utilizing jump assessing equipment.

## Figures and Tables

**Table 1:** Summary statistics for jump heights, contact-time, and reactive strength index across all devices, jumps, and sessions.

Variable	Mean	SD	Minimum	Maximum	%Δ vs. Force-Place	Effect Size (95% CI) vs. Force-Plate
**Force-plate**						
SJ height (cm)	28.4	8.0	13.8	42.4	N/A	N/A
CMJ height (cm)	30.6	8.5	12.1	43.3	N/A	N/A
DJ height (cm)	26.3	7.9	9.4	43.3	N/A	N/A
DJ contact-time (ms)	332	86.9	201	553	N/A	N/A
RSI	0.84	0.30	0.27	1.50	N/A	N/A
**Mo-Cap**						
SJ height (cm)	27.7	7.9	12.2	42.8	−2.5%	−0.09 (−0.19, 0.37)
CMJ height (cm)	30.2	8.8	13.9	44.7	−1.3%	−0.05 (−0.32, 0.23)
DJ height (cm)	26.2	8.3	8.9	42.7	−0.4%	−0.01 (−0.29, 0.27)
DJ contact-time (ms)	346	87.5	205	584	+4.2%	0.16 (−0.12, 0.44)
RSI	0.80	0.30	0.28	1.46	−4.8%	−0.13 (−0.41, 0.14)
**G-Flight**						
SJ height (cm)	32.5	7.3	18.0	45.6	+14.4%	0.53 (−0.25, 0.82)
CMJ height (cm)	33.8	8.1	17.8	48.5	+10.6%	0.38 (−0.11, 0.67)
DJ height (cm)	27.6	8.4	8.7	46.8	+4.9%	0.16 (−0.12, 0.44)
DJ contact-time (ms)	339	87.0	224	578	+2.1%	0.08 (−0.20, 0.36)
RSI	0.85	0.29	0.26	1.42	+1.2%	0.03 (−0.24, 0.31)
**PUSH**						
SJ height (cm)	32.4	6.9	19.3	44.8	+14.1%	0.53 (−0.25, 0.82)
CMJ height (cm)	34.4	7.2	19.8	45.3	+12.4%	0.48 (−0.20, 0.76)
DJ height (cm)	30.2	7.4	14.7	45.8	+14.8%	0.51 (−0.23, 0.79)
DJ contact-time (ms)	322	84.8	191	514	−3.0%	−0.12 (−0.39, 0.16)
RSI	0.78	0.24	0.27	1.38	−7.1%	−0.22 (−0.50, 0.06)

SD = standard deviation. SJ = squat jump. CMJ = countermovement jump. DJ = drop jump. RSI = reactive strength index. RSI = jump height in centimeters divided by contact-time in milliseconds. Effect size = Cohen’s *d*. CI = confidence interval. N/A = not applicable.

**Table 2:** Intraclass correlation coefficient (ICC), typical error of measurement (TEM), and coefficient of variance (CV) for force-plate, Mo-Cap, G-Flight, and Push for each jump, contact-time, and RSI of three repetitions of each test session.

Variable	ICC	95% CI	TEM Session 1	TEM Session 2	TEM Session 3	CV Session 1	CV Session 2	CV Session 3
**Force-plate**								
SJ height	0.994	0.990–0.997	1.4 ± 0.9	1.0 ± 0.5	1.4 ± 0.7	4.8 ± 2.8	3.8 ± 2.1	5.0 ± 2.8
CMJ height	0.994	0.989–0.997	1.2 ± 0.8	1.0 ± 0.6	1.3 ± 0.6	3.9 ± 2.6	3.4 ± 1.9	4.1 ± 2.0
DJ height	0.991	0.984–0.995	1.0 ± 0.6	1.2 ± 0.4	1.8 ± 0.8	4.3 ± 2.7	5.1 ± 3.2	7.8 ± 5.0
DJ contact-time	0.984	0.973–0.991	21.0 ± 9	22.1 ± 15	16.0 ± 9	6.2 ± 3.0	6.7 ± 4.3	5.0 ± 2.5
RSI	0.914	0.851–0.953	0.05 ± 0.03	0.06 ± 0.04	0.07 ± 0.04	7.0 ± 3.5	7.6 ± 6.3	9.7 ± 6.0
**Mo-Cap**								
SJ height	0.99	0.982–0.995	1.1 ± 0.7	1.4 ± 0.9	1.1 ± 0.8	4.0 ± 2.4	6.1 ± 5.4	3.9 ± 2.7
CMJ height	0.979	0.963–0.989	1.6 ± 1.1 *	1.6 ± 1.2	2.2 ± 1.7	5.2 ± 3.0	5.5 ± 3.2 *	7.1 ± 4.5
DJ height	0.985	0.974–0.992	1.2 ± 0.5	1.6 ± 1.0	1.8 ± 1.0	5.2 ± 3.3	7.9 ± 6.9	8.3 ± 6.4
DJ contact-time	0.979	0.963–0.989	21 ± 9	19 ± 9	23 ± 13	5.7 ± 1.9	5.6 ± 2.1	7.2 ± 3.5
RSI	0.980	0.965–0.989	0.06 ± 0.03	0.06 ± 0.03	0.08 ± 0.05	9.0 ± 4.5	9.0 ± 5.9	11.1 ± 7.6
**G-Flight**								
SJ height	0.983	0.970–0.991	1.7 ± 0.8	1.4 ± 1.1	1.6 ± 0.6	5.6 ± 3.3	4.6 ± 3.8	5.0 ± 1.7
CMJ height	0.958	0.928–0.977	2.6 ± 1.4 *	2.7 ± 2.0 *	2.0 ± 0.9 *	8.1 ± 4.8 *	9.0 ± 7.8 *	5.9 ± 1.8 *
DJ height	0.978	0.962–0.988	1.8 ± 1.2 *	2.1 ± 1.3 *	1.8 ± 1.0	7.3 ± 5.7 *	8.9 ± 6.4 *	7.6 ± 6.6
DJ contact-time	0.974	0.954–0.986	21 ± 12	23 ± 11	23 ± 13	5.8 ± 3.6	6.9 ± 2.8	7.1 ± 4.3
RSI	0.961	0.932–0.979	0.08 ± 0.04 *	0.09 ± 0.05	0.10 ± 0.04	10.6 ± 4.3 *	11.0 ± 6.3 *	12.6 ± 6.8
**PUSH**								
SJ height	0.982	0.967–0.991	1.2 ± 1.0	1.3 ± 0.9	1.8 ± 0.9	3.6 ± 2.7	3.9 ± 2.5	5.6 ± 2.8
CMJ height	0.986	0.975–0.993	1.4 ± 0.7	1.4 ± 0.8 *	1.1 ± 0.5	4.0 ± 1.8	4.1 ± 2.5	3.1 ± 1.3
DJ height	0.980	0.963–0.990	1.6 ± 1.1 *	1.4 ± 0.4	2.2 ± 0.9	5.4 ± 3.8	5.1 ± 2.2	7.5 ± 3.3
DJ contact-time	0.974	0.953–0.987	21 ± 13	16 ± 8	23 ± 17	6.1 ± 2.8	5.3 ± 2.5	7.6 ± 5.8
RSI	0.963	0.932–0.981	0.06 ± 0.03	0.05 ± 0.04	0.10 ± 0.06	9.0 ± 4.4	6.9 ± 4.9	13.5 ± 8.8

CI = confidence interval. * indicates a significant difference with force-plate on *p* < 0.05 level. SJ = squat jump. CMJ = countermovement jump. DJ = drop jump. RSI = reactive strength index. Contact-time is recorded in milliseconds. RSI = jump height in centimeters divided by contact-time in milliseconds.
